# Prevalence and factors influencing disability and cognitive impairment among empty nesters and non-empty nesters in Guangdong, China: a cross-sectional study

**DOI:** 10.3389/fpubh.2025.1545497

**Published:** 2025-03-27

**Authors:** Jinhua Guo, Yi Yang, Lixia Lin, Yi Zhang, Tiemei Shen

**Affiliations:** ^1^Guangdong Cardiovascular Institute, Guangdong Provincial People’s Hospital, Guangdong Academy of Medical Sciences, Southern Medical University, Guangzhou, Guangdong, China; ^2^Department of Nursing, Guangdong Provincial People’s Hospital, Guangdong Academy of Medical Sciences, Southern Medical University, Guangzhou, Guangdong, China

**Keywords:** empty nest, older adults, disability, cognitive impairment, root cause analysis

## Abstract

**Background:**

Disability and cognitive impairment affect the physical and mental health of older adult individuals and also impose a heavy burden on families and society. As a threat to their health, the growing trend of empty nesting among older adult individuals is attracting widespread attention.

**Objective:**

To investigate the status of disability and cognitive impairment among empty nesters and non-empty nesters in Guangdong Province and to analyze the differences in their influencing factors to provide a scientific basis for the prevention and control of disability among empty nesters and non-empty nesters.

**Methods:**

Using the stratified random cluster sampling method, we recruited 5,603 individuals older adults 60 years and older from 21 cities in Guangdong Province in southern China, comprising 1,512 empty nesters and 4,091 non-empty nesters. Physical function and cognitive impairment were assessed with the ability to perform basic activities of daily living (BADLs) and scores on the Mini-Mental State Examination (MMSE). Binary logistic regression was performed to analyze the risk factors in the two groups.

**Results:**

The prevalence of disability and cognitive impairment among empty nesters was significantly higher than that in non-empty nesters (40.15% vs. 35.74, 27.51% vs. 23.52%, respectively). Common influencing factors for the occurrence of disability in empty nesters and non-empty nesters were as follows. Binary logistic regression showed that the average monthly household income of empty nesters was 2000–400 RMB (OR: 1.476, 95% CI 1.019, 2.138) and for non-empty nesters was 2000–400 RMB (OR: 1.353, 95% CI 1.048, 1.747). Many study subjects took more than four types of medications (empty nesters: OR: 3.166, 95% CI 1.940, 5.169; non-empty nesters: OR: 2.660, 95% CI 1.957, 3.615). Both populations reported family support (empty nesters: OR: 1.487, 95% CI 1.064, 2.077 and non-empty nesters: OR: 1.341, 95% CI 1.106, 1.626), depression (empty nesters: OR: 1.710, 95% CI 1.104, 2.471 and non-empty nesters: OR: 1.990, 95% CI 1.524, 2.599), and anxiety (yes: OR: 1.652, 95% CI 1.104, 2.471), which was an influential factor specific to the occurrence of disability in empty nesters (*p* < 0.05). Education level, residence, and depression among empty nesters (OR: 3.111, 95% CI 2.059, 4.701) and non-empty nesters (OR: 1.892, 95% CI 1.461, 2.451) were common influencing factors for the occurrence of cognitive impairment in both groups (*p* < 0.05). Category 1 medications were an influential factor specific to cognitive impairment among empty nesters (OR: 1.564, 95% CI 1.072, 2.282; *p* < 0.05); and coronary heart disease was an influencing factor specific to cognitive impairment among non-empty nesters (OR: 1.319, 95% CI 1.046, 1.663; *p* < 0.05).

**Conclusion:**

The study indicated that empty nesters had a higher prevalence of disability and cognitive impairment than non-empty nesters. The influencing factors were different between empty nesters and non-empty nesters. Low and middle incomes, multiple medication use, family support, and depression were the common influencing factors for the occurrence of disability among empty nesters and non-empty nesters, while anxiety was the unique influencing factor for disability among empty nesters. Literacy level, place of residence, and depression were the common influencing factors for cognitive impairment among empty nesters and non-empty nesters. Multiple medications were an influencing factor specific to cognitive impairment among empty nesters. Coronary heart disease was an influencing factor specific to cognitive impairment in non-empty nesters. Therefore, when intervening in older adults individuals with disability and cognitive impairment, different measures should be taken according to whether they are empty nesters or not.

## Introduction

With the accelerated modernization process, population aging has become a worldwide phenomenon. China now has the largest older adult population in the world, and the aging process will further accelerate. According to the annual data of the National Bureau of Statistics of China (CNBS), in 2020, the population over 60 years of age was 264 million, and that over 65 years of age was 191 million (1). It is estimated that the total number of older adult people in China will be approximately 300 million in 2025 and will reach more than 400 million in 2033. By 2053, China will be home to an older adult population of 483 million, representing two-fifths of the older adult people in Asia and a quarter of the world’s older adult population (2). Along with the aging of Chinese society and the change in the concept of marriage and childbirth, the growing older adults population, combined with smaller family size, simpler family structure, and discrete living patterns, is driving the growing number of older adults empty-nest families, which are composed of only older adults couples or older adults singles living alone (3). Empty nesters are older adult individuals who have no children, have children who live elsewhere, live with a spouse or live alone (4). Studies have shown that by 2025, the number of empty-nest older adults in our country will reach 113 million, with the figure expected to exceed 200 million, and the proportion of empty-nest families is projected to reach 90% (5, 6).

The World Health Organization defines disability as a collective term for impairment, activity limitation and participation restriction. It is an important indicator of an individual’s health (7). Along with aging and advanced age, physical function and health status of older adult individuals decline, and disability and cognitive impairment become major health problems. Cognitive impairment occurs on a continuum from age-related subjective cognitive decline to mild cognitive impairment, often leading to dementia (8). Empty nesters are a special group of older adult individuals, and they have more needs for life care, health care, and spiritual comfort (9–11). Several studies have noted that there are differences between empty nesters and non-empty nesters in terms of psychological and social support, and it is worth paying attention to whether there are differences in the disabling conditions of the two groups (12, 13). It is expected that by 2050, the number of disabled older adults and partially disabled older adults individuals in China will reach 68 million (14). The problem of disability not only affects the physical and mental health of the individual but also places a burden on families and society.

Previous studies have shown that empty nesters are in a miserable situation in terms of quality of life (15), social support (16), and depression (17). Does empty nesting lead to disability and cognitive impairment in the older adults? Several studies have reported on disability and dementia in older adults. In the last 3 years, several studies have been conducted on the current situation of older people with MCI or dementia, and the estimated prevalence ranges from 1.2 to 23.2% (18–20), which is nearly 15% in China (21, 22). However, relatively few studies have addressed differences in disability, cognitive impairment and their associated factors between empty nesters and non-empty nesters. Some studies have shown that empty nesters have lower levels of disability than non-empty nesters (23). However, no difference has been observed in the cognitive impairment of empty nesters and non-empty nesters. Based on the above, we have expanded the study area and increased the number of respondents to explore whether there are differences in the occurrence of disability and cognitive impairment between empty nesters and non-empty nesters. In addition, this study comparatively evaluates the disability and cognitive impairment of empty nesters and non-empty nesters, explores the risk factors for empty nesters and non-empty nesters, and provides a valid theoretical basis for interventions targeting empty nesters and non-empty nesters.

## Methods

### Design and participants

The selected survey site was Guangdong Province in southern China. A stratified random whole-group sampling method was used to recruit older adults people (older adults 60 and older) from 21 cities in Guangdong Province: Guangzhou, Shenzhen, Zhuhai, Dongguan, Foshan, Zhongshan, Huizhou, Shantou, Jiangmen, Zhanjiang, Zhaoqing, Meizhou, Maoming, Yangjiang, Qingyuan, Shaoguan, Jieyang, Shanwei, Chaozhou, Heyuan and Yunfu. The sampling method was as follows: In the first stage, we numbered the districts (counties) of the 21 cities in the order of the municipal districts (counties) under their jurisdiction per the government website. In the second stage, a random number table was used to select two districts (counties) in each city, and then two communities (administrative villages) were selected from each district (county) in the same way. In the fourth stage, we extracted two neighborhoods (villages) from each community (administrative village). Elders in the two neighborhoods (natural administrative villages) who met the criteria were included in this study. The inclusion criteria were (1) ≥60 years or older, (2) normal communication without barriers, and (3) informed consent and volunteering for this study.

All participants were informed of the purpose and methods of the study at the time of recruitment and were assured of their right to refuse participation, and all voluntarily signed an informed consent form prior to participation. This study was approved by the Ethics Committee of Guangdong Provincial People’s Hospital (Ethics No. KY-Z-2021-690-01).

According to the sample size estimation method (24), the sample size is the number of variables of 5 to 10 times, there were 29 variables in this study, and considering 10% of invalid question volumes, resulting in a sample size of 160–319 cases. The study was conducted between January 2022 and April 2022 and involved 6,000 older people, 5,603 of whom completed the questionnaire for an effective rate of 93.38%.

### Assessments and procedure

Data collection was conducted through the WeChat applet ‘Jingyice platform on the functional assessment of older adults. First, we contacted the relevant leaders of the selected hospitals and communities to obtain permission to conduct the survey. Each hospital and community had dedicated interviewers responsible for collecting information on each specific study area. Interviewers in the corresponding communities were responsible for conducting household surveys. To ensure uniformity in the survey process, online training on questionnaire interpretation and methodology was organized, and a preliminary survey was conducted prior to the formal survey. Interviewers used a uniform guide to introduce the purpose and content of the study and used one-on-one, face-to-face dialog to obtain information after informed consent. Respondents were asked about family members or careers if they were unable to communicate directly with the interviewer due to speech or hearing impairments. Each data point was kept in a secure file that could only be accessed by authorized personnel. The questionnaire consisted of three sections: a self-administered basic information section, basic activities of daily life (BADLs) and Mini-Mental State Examination (MMSE).

The homemade basic information questionnaire was used to measure sociodemographic characteristics, including age, gender, BMI, number of children, smoking, drinking, annual physical examination, weekly social activities and weekly exercise. Family or social support was assessed around support from family or care from family or friends, cardiovascular or cerebrovascular diseases, hypertension, coronary heart disease, diabetes, type of medication, hearing disorder, vision disorder and walking impairment.

This population-based cross-sectional investigation systematically operationalized the World Health Organization (WHO) standardized assessment protocol for Basic Activities of Daily Living (BADL), as codified in the International Classification of Functioning (25), BADLs refer to the basic movements and self-care activities performed in hospitals or homes, which included eight items (eating, bathing, combing hair, dressing, controlling urine, controlling excrement, walking and walking up and down stairs) (25). There are eight self-assessed/other-assessed items in this scale. The ability to perform these measures of self-care were scored as follows: 100 points, good ability to perform activities of daily living without the help of others; 61 to 99 points, mild dysfunction (able to complete some BADLs independently but need some help); 41 to 60 points, moderate dysfunction (need much help to complete BADLs; ≤ 40 points), severe dysfunction (all or most BADLs cannot be completed independently).

We employed the Chinese version of the Mini-Mental State Examination to evaluate cognitive impairment. The scale, which has a maximum score of 30, was developed by Folstein et al. (26) in 1975. The MMSE assesses patients in seven main areas: temporal orientation, place orientation, immediate memory, delayed memory, numeracy and attention, verbal ability, and visuospatial ability. The scale is one of the most influential screening tools for cognitive impairment available. The Chinese version of the MMSE, revised and adapted by Zhang Mingyuan, is commonly used for clinical screening of patients with cognitive impairment in China, with a Cronbach alpha coefficient of 0.833 and a retest reliability of 0.924. A score of ≤17 for illiteracy, ≤20 for primary school literacy and ≤ 24 for junior high school and more indicates cognitive impairment (26, 27).

### Statistical analysis

SPSS 26.0 was used for statistical analysis. Qualitative variables were statistically described using frequencies, quantitative variables that conformed to a normal distribution were statistically described using means (standard deviations), and quantitative variables that did not conform to a normal distribution were statistically described using medians (four-score intervals). Comparisons between groups were made using the t test for quantitative variables and the chi-square test for qualitative variables. Count data were expressed as relative numbers, and group comparisons were made using the χ^2^ test. Dichotomous logistic stepwise regression analysis was used to explore the factors influencing the occurrence of disability and cognitive impairment among empty nesters and non-empty nesters. Two-sided test level *α* = 0.05.

## Results

### Basic characteristics

A total of 5,603 older adult individuals were recruited for our study, comprising 1,512 (26.99%) empty nesters and 4,091 (73.01%) non-empty nesters. The mean age was 71.38 ± 7.64. Among the participants, 2,657 were males (47.42%), and 2,946 were females (52.58%). The participants reported the following demographic information: In rural areas, 41.51% reported living close to a neighbor. Most participants had no more than a primary school (61.95%), an income less than RMB4000 (55.56%), received financial support from their children (69.52%), and lived with a partner (81.69%). Statistically significant differences in basic characteristics among empty and non-empty nesters were found for gender, average monthly household income, primary source of income, education level, marital status, place of residence, mode of health insurance, coronary heart disease, type of medication taken, smoking, alcohol consumption, physical examination every 10 years, weekly participation in physical activities, weekly participation in social activities, family support, family or friend care, anxiety, depression, and vision. Among the participants, 607 empty nesters (40.15%) and 1,462 non-empty nesters (35.74%) had physical function impairment. The prevalence of physical function impairment was 36.93% among all participants and higher in the empty nesters than in non-empty nesters (40.15% vs. 35.74%; *p* < 0.05, Table 1). Among the participants, 416 empty nesters (27.51) and 962 (23.52%) non-empty nesters had cognitive impairment. The prevalence of cognitive impairment was 24.59% in all participants and was higher in the empty nesters than in non-empty nesters (27.51% vs. 23.52%; *p* < 0.05, Table 1).

**Table 1:** Comparison of demographics and physical and cognitive impairment among empty nesters and non-empty nesters.

Characteristics	Empty nest [n (%)]	Non-empty nest [n (%)]	*t* or *χ*^2^	*p-*value
Age, years old [Mean (Std)]	71.66 (7.67)	71.24 (7.43)	1.669	0.095
BMI, kg/m^2^ [Mean (Std)]	23.07 (3.72)	22.99 (3.55)	0.766	0.444
Sex			19.357	<0.001
Male	790 (52.25)	1867 (45.64)		
Female	722 (47.75)	2,224 (54.36)		
Region			57.149	<0.001
Rural	720 (47.62)	1,606 (39.26)		
County or Town	245 (16.20)	610 (14.91)		
Small and medium-sized cities	213 (14.09)	564 (13.79)		
Big Cities	334 (22.09)	1,311 (32.05)		
Religion			0.187	0.666
Yes	117 (7.74)	331 (8.09)		
No	1,395 (92.26)	3,760 (91.90)		
Family income per month, CNY			117.370	<0.001
<2000	425 (28.11)	683 (16.70)		
2000–4,000	554 (36.64)	1,451 (35.47)		
4,000–6,000	274 (18.12)	892 (21.80)		
>6,000	259 (17.13)	1,065 (26.03)		
Source of income			160.849	<0.001
Acquiring from children	874 (57.80)	3,021 (73.85)		
Retirement income	450 (29.76)	862 (21.07)		
Labor income	188 (12.44)	208 (5.08)		
Education level			20.236	<0.001
Illiteracy	302 (19.97)	813 (19.87)		
Primary school and below	602 (39.81)	1754 (42.87)		
Junior high school	297 (19.64)	828 (20.24)		
Senior high school	233 (15.41)	578 (14.13)		
College and above	78 (5.16)	118 (2.88)		
Marital status			14.616	<0.001
With partner	1,186 (78.44)	3,391 (82.89)		
No partner	326 (21.56)	700 (17.11)		
Place of residence			57.149	<0.001
Villages	720 (47.62)	1,606 (39.26)		
Counties or towns	245 (16.20)	610 (14.91)		
Small or middle-sized cities	213 (14.09)	564 (13.79)		
Large-sized cities	334 (22.09)	1,311 (32.05)		
Type of insurance			11.431	0.003
Rural medical service	877 (58.00)	2,575 (62.94)		
Urban medical service	527 (34.85)	1,253 (30.63)		
Free medical service	108 (7.15)	263 (6.43)		
Cardiovascular or cerebrovascular diseases			3.043	0.081
Yes	911 (60.25)	2,359 (57.66)		
No	6,016 (39.75)	1732 (42.34)		
Hypertension			0.549	0.459
Yes	639 (42.26)	1,684 (41.16)		
No	873 (57.74)	2,407 (58.84)		
Coronary heart disease			8.439	0.004
Yes	296 (19.58)	666 (16.28)		
No	1,216 (80.42)	3,425 (83.72)		
Diabetes			1.697	0.193
Yes	303 (20.04)	757 (18.50)		
No	1,209 (79.96)	3,334 (81.50)		
Type of medication			15.568	0.004
None	538 (35.58)	1,617 (39.53)		
1	279 (18.45)	798 (19.51)		
2	300 (19.84)	793 (19.38)		
3	176 (11.64)	409 (10.00)		
>4	219 (14.48)	474 (11.59)		
Drinking			29.680	<0.001
Never	1,134 (75.00)	3,316 (81.06)		
Occasional	196 (12.96)	351 (8.58)		
Always	182 (12.04)	424 (10.36)		
Smoking			24.408	<0.001
Never	1,002 (66.27)	2,978 (72.79)		
Occasional	250 (16.53)	510 (12.47)		
Always	260 (17.20)	603 (14.74)		
Annual physical examination last 10 years			32.749	<0.001
Yes	574 (37.96)	1903 (46.52)		
No	938 (62.04)	2,188 (53.48)		
Weekly social activities, days			17.260	0.001
Never	657 (43.45)	1,656 (40.48)		
1–3	444 (29.37)	1,087 (26.57)		
4–6	52 (3.44)	176 (4.30)		
Every day	359 (23.74)	1,172 (28.65)		
Weekly exercise, days			24.269	<0.001
Never	599 (39.62)	1,343 (32.83)		
1–3	345 (22.82)	1,005 (24.57)		
4–6	52 (3.44)	191 (4.67)		
Every day	516 (34.12)	1,552 (37.93)		
Support from family			91.008	<0.001
Yes	663 (43.85)	2,379 (58.15)		
No	849 (56.15)	1712 (41.85)		
Care from family or friends			148.931	<0.001
Yes	1,076 (71.16)	3,494 (85.41)		
No	436 (28.84)	597 (14.59)		
Anxiety			5.579	0.018
Yes	452 (29.89)	1,093 (26.72)		
No	1,060 (70.11)	2,998 (73.28)		
Depression			9.684	0.002
Yes	422 (27.91)	976 (23.86)		
No	1,090 (72.09)	3,115 (76.14)		
Hearing			3.330	0.068
Normal	1,024 (67.72)	2,874 (70.25)		
Abnormal	488 (32.28)	1,217 (29.75)		
Vision			10.345	0.001
Normal	925 (61.18)	2,693 (65.83)		
Abnormal	587 (38.82)	1,398 (34.17)		
Physical function impairment			7.254	0.007
Yes	607 (40.15)	1,462 (35.74)		
No	905 (59.85)	2,609 (64.26)		
Cognitive impairment			9.516	0.002
Yes	416 (27.51)	962 (23.52)		
No	1,096 (72.49)	3,129 (76.48)		

### Analysis of factors affecting the disability of empty nesters

The dependent variable was whether the empty nesters had a combined disability or not (assigned values were no = 0, yes = 1), and the statistically significant basic characteristics in Table 1 were used as independent variables in a dichotomous logistic stepwise regression analysis. Results indicated that a family income per month between 2000 to 4000RMB (OR: 1.476, 95% CI: 1.019, 2.138) and a retirement income/pension decreased the risk of physical function impairment (OR: 0.679, 95% CI: 0.469, 0.982). Taking four or more medications (OR: 3.166, 95% CI: 1.940, 5.169) was associated with a higher risk of physical function impairment. Annual physical examinations for the past 10 years and being cared for by family or friends were associated with a lower risk of physical function impairment. Older adult people without weekly activities were more likely to face physical function impairment than those participating in activities 1–3 days per week or every day. Support from family (OR: 1.487, 95% CI: 1.064, 2.077), anxiety (OR: 1.652, 95% CI: 1.104, 2.471), depression (OR: 1.710, 95% CI: 1.120, 2.612) were also correlated with a higher risk of physical functional impairment. Multivariate logistic regression analysis of risk factors for physical impairment among adults older adults 60 years or older is presented in Table 2.

**Table 2:** Dichotomous logistic regression analysis of factors affecting the disability of empty nesters.

Influencing factors	*β (SE)*	Wald χ^2^ value	*p* value	Adjusted *OR* (*95%*CI)
Gender	0.166 (0.173)	0.921	0.337	1.181 (0.841,1.658)
Family income per month, CNY				
<2000	as ref			
2000–4,000	0.390 (0.189)	4.250	0.039	1.476 (1.019,2.138)
4,000–6,000	0.120 (0.241)	0.248	0.619	1.127 (0.703,1.807)
>6,000	0.329 (0.250)	1.729	0.189	1.389 (0.85,2.268)
Source of income				
Acquiring from children	as ref			
Retirement income	−0.388 (0.189)	4.222	0.040	0.679 (0.469,0.982)
Labor income	−0.097 (0.256)	0.143	0.705	0.908 (0.549,1.500)
Education level				
Illiteracy	as ref			
Primary school and below	−0.177 (0.197)	0.812	0.367	0.837 (0.569,1.232)
Junior high school	−0.71 (0.13)	0.881	0.348	0.798 (0.499,1.278)
Senior high school	−0.75 (0.15)	0.133	0.716	0.905 (0.529,1.548)
College and above	−0.49 (0.24)	0.093	0.760	0.885 (0.405,1.936)
Marital status	0.044 (0.187)	0.057	0.812	1.045 (0.725,1.507)
Place of residence				
Villages	as ref			
Counties or towns	−0.132 (0.219)	0.362	0.547	0.876 (0.570,1.347)
Small or middle-sized cities	0.129 (0.248)	0.271	0.603	1.138 (0.700,1.851)
Large-sized cities	−0.193 (0.223)	0.744	0.388	0.825 (0.532,1.278)
Coronary heart disease	0.115 (0.191)	0.365	0.546	1.122 (0.772,1.631)
Type of insurance				
Rural medical service	as ref			
Urban medical service	0.270 (0.196)	1.889	0.169	1.309 (0.891,1.924)
Free medical service	0.291 (0.297)	0.963	0.327	1.338 (0.748,2.396)
Type of medication				
None	as ref			
1	0.369 (0.210)	3.097	0.078	1.446 (0.959,2.182)
2	0.770 (0.206)	13.934	<0.001	2.159 (1.441,3.234)
3	0.482 (0.247)	3.816	0.051	1.619 (0.998,2.625)
>4	1.153 (0.250)	21.254	<0.001	3.166 (1.940,5.169)
Drinking				
Never	as ref			
Occasional	0.387 (0.248)	2.420	0.120	1.472 (0.904,2.395)
Always	0.265 (0.247)	1.150	0.284	1.303 (0.803,2.113)
Smoking				
Never	as ref			
Occasional	−0.275 (0.227)	1.473	0.225	0.759 (0.487,1.185)
Always	−0.149 (0.234)	0.409	0.523	0.861 (0.545,1.362)
Annual physical examination last 10 years	−0.350 (0.169)	4.293	0.038	0.705 (0.506,0.981)
Weekly social activities, days				
Never	as ref			
1–3	−0.260 (0.179)	2.113	0.146	0.771 (0.542,1.095)
4–6	−0.526 (0.432)	1.483	0.223	0.591 (0.253,1.378)
Every day	−0.389 (0.208)	3.511	0.061	0.678 (0.451,1.018)
Weekly exercise, days				
Never	as ref			
1–3	−0.662 (0.195)	11.563	0.001	0.516 (0.352,0.756)
4–6	−0.843 (0.431)	3.827	0.050	0.430 (0.185,1.002)
Every day	−0.769 (0.194)	15.784	<0.001	0.463 (0.317,0.677)
Care from family or friends	−0.577 (0.188)	9.470	0.002	0.562 (0.389,0.811)
Support from family	0.396 (0.171)	5.369	0.020	1.487 (1.064,2.077)
Vision	0.153 (0.152)	1.020	0.313	1.165 (0.866,1.568)
Anxiety	0.502 (0.206)	5.967	0.015	1.652 (1.104,2.471)
Depression	0.537 (0.216)	6.171	0.013	1.710 (1.120,2.612)

### Analysis of factors affecting the cognitive impairment of empty nesters

The dependent variable was whether the empty nesters had a combined cognitive impairment or not (assigned values were no = 0, yes = 1), and the statistically significant basic characteristics in Table 1 were used as independent variables in a dichotomous logistic stepwise regression analysis. Results indicated that education level influenced the risk of cognitive impairment, and this risk was higher among those with junior high school (OR: 4.744, 95% CI: 3.246, 6.931), senior high school (OR: 3.059, 95% CI: 2.045, 4.576) or college (OR: 2.688, 95%CI: 1.529, 4.655) education levels. Living in small or medium-sized cities (OR: 2.167, 95% CI: 1.392, 3.374) was associated with a higher risk of cognitive impairment. Annual physical examination for the past 10 years and being cared for by family or friends were associated with a lower risk of physical function impairment. Older adults people who did not participate in social activities weekly were more likely to face cognitive impairment than those who participated in social activities 1–3 days per week (OR: 0.544, 95% CI: 0.358, 0.769). Care from family and friends reduced the incidence of cognitive impairment (OR: 0.640, 95% CI: 0.451, 0.907). Depression was also correlated with a higher risk of physical functional impairment (OR: 3.111, 95% CI: 2.059, 4.701). Multivariate logistic regression analysis of risk factors for cognitive impairment among adults older adults 60 years or older is presented in Table 3.

**Table 3:** Dichotomous logistic regression analysis of factors affecting cognitive impairment of empty nesters.

Influencing factors	*β (SE)*	Wald χ^2^ value	*p* value	Adjusted *OR* (*95%*CI)
Gender	0.272 (0.162)	2.831	0.092	1.313 (0.956,1.802)
Family income per month, CNY				
<2000	as ref			
2000–4,000	−0.105 (0.183)	0.328	0.567	0.901 (0.630,1.288)
4,000–6,000	−0.385 (0.233)	2.278	0.099	0.681 (0.431,1.074)
>6,000	−0.317 (0.239)	1.751	0.186	0.729 (0.456,1.165)
Source of income				
Acquiring from children	as ref			
Retirement income	−0.274 (0.176)	2.439	0.118	0.760 (0.539,1.072)
Labor income	0.019 (0.239)	0.006	0.938	1.019 (0.638,1.627)
Education level				
Illiteracy	as ref			
Primary school and below	0.086 (0.188)	0.208	0.649	1.089 (0.754,1.575)
Junior high school	1.557 (0.194)	64.717	<0.001	4.744 (3.246,6.931)
Senior high school	1.118 (0.205)	29.600	<0.001	3.059 (2.045,4.576)
College and above	0.981 (0.284)	11.948	0.001	2.688 (1.529,4.655)
Marital status	−0.074 (0.177)	0.173	0.678	0.929 (0.657,1.314)
Place of residence				
Villages	as ref			
Counties or towns	0.238 (0.214)	1.231	0.267	1.269 (0.833,1.932)
Small or middle-sized cities	0.773 (0.226)	11.720	0.001	2.167 (1.392,3.374)
Large-sized cities	0.353 (0.207)	2.906	0.088	1.423 (0.949,2.136)
Coronary heart disease	0.208 (0.183)	1.290	0.256	1.231 (0.860,1.763)
Type of insurance				
Rural medical service	as ref			
Urban medical service	−0.058 (0.180)	0.106	0.745	0.943 (0.663,1.341)
Free medical service	−0.063 (0.273)	0.053	0.818	0.939 (0.549,1.064)
Type of medication				
None	as ref			
1	0.447 (0.193)	5.36	0.020	1.564 (1.072,2.282)
2	−0.098 (0.204)	0.228	0.633	0.907 (0.608,1.353)
3	−0.131 (0.241)	0.295	0.587	0.877 (0.547,1.407)
>4	0.058 (0.238)	0.060	0.806	1.060 (0.665,1.689)
Drinking				
Never	as ref			
Occasional	0.066 (0.228)	0.083	0.773	1.068 (0.683,1.669)
Always	−0.092 (0.248)	0.137	0.711	0.912 (0.562,1.483)
Smoking				
Never	as ref			
Occasional	−0.126 (0.220)	0.329	0.566	0.881 (0.572,1.357)
Always	−0.223 (0.227)	0.969	0.325	0.800 (0.513,1.247)
Annual physical examination last 10 years	−0.119 (0.159)	0.562	0.453	0.888 (0.651,1.212)
Weekly social activities, days				
Never	as ref			
1–3	−0.609 (0.176)	11.898	0.001	0.544 (0.385,0.769)
4–6	−0.819 (0.418)	3.839	0.050	0.441 (0.194,1.000)
Every day	−0.291 (0.198)	2.162	0.141	0.747 (0.507,1.102)
Weekly exercise, days				
Never	as ref			
1–3	−0.031 (0.195)	0.026	0.872	0.969 (0.661,1.420)
4–6	−0.183 (0.417)	0.192	0.661	0.833 (0.38,1.886)
Every day	0.292 (0.189)	2.390	0.122	1.339 (0.925,1.937)
Care from family or friends	−0.447 (0.178)	6.285	0.012	0.640 (0.451,0.907)
Support from family	−0.124 (0.160)	0.606	0.436	0.883 (0.646,1.208)
Vision	0.017 (0.147)	0.013	0.313	1.165 (0.866,1.568)
Anxiety	−0.044 (0.204)	0.046	0.803	0.957 (0.642,1.427)
Depression	1.135 (0.211)	229.056	<0.001	3.111 (2.059,4.701)

### Analysis of factors affecting the disability of non-empty nesters

The dependent variable was whether the non-empty nesters had a combined disability or not (assigned values were no = 0, yes = 1), and the statistically significant basic characteristics in Table 1 were used as independent variables in a dichotomous logistic stepwise regression analysis. Results indicated that family income per month between RMB 2000 to 4,000 (OR:1.476, 95% CI: 1.019, 2.138) and retirement income/pension between RMB 4000 to 6,000￥(OR:1.481, 95% CI: 1.105, 1.985) decreased the risk of physical function impairment (OR: 0.658, 95% CI: 0.514, 0.844), education level, drinking, smoking, annual physical examination for the past 10 years, participating in social activities 1–3 days per week or every day, activities every day, care from family or friends were negatively associated with physical impairment in non-empty nesters. Medication, family support and depression were positively associated with physical impairment in non-empty nesters. Multivariate logistic regression analysis of risk factors for physical impairment among adults older adults 60 years or older is presented in Table 4.

**Table 4:** Dichotomous logistic regression analysis of factors affecting the disability of non-empty nesters.

Influencing factors	*β (SE)*	Wald χ^2^ value	*p* value	Adjusted *OR* (*95%*CI)
Gender	−0.029 (0.104)	0.079	0.779	0.971 (0.791,1.192)
Family income per month, CNY				
<2000	as ref			
2000–4,000	0.303 (0.130)	5.348	0.020	1.353 (1.048,1.747)
4,000–6,000	0.393 (0.149)	6.915	0.009	1.481 (1.105,1.985)
>6,000	0.027 (0.147)	0.035	0.852	1.028 (0.771,1.371)
Source of income				
Acquiring from children	as ref			
Retirement income	−0.418 (0.127)	10.865	0.001	0.658 (0.514,0.844)
Labor income	−0.223 (0.204)	1.191	0.275	0.800 (0.536,1.194)
Education level				
Illiteracy	as ref			
Primary school and below	−0.421 (0.117)	13.036	<0.001	0.656 (0.522,0.825)
Junior high school	−0.700 (0.147)	22.573	<0.001	0.497 (0.372,0.663)
Senior high school	−0.877 (0.171)	26.337	<0.001	0.416 (0.298,0.582)
College and above	−0.474 (0.300)	2.490	0.115	0.623 (0.346,1.122)
Marital status	0.187 (0.117)	2.538	0.111	1.205 (0.958,1.516)
Place of residence				
Villages	as ref			
Counties or towns	0.234 (0.134)	3.044	0.081	1.264 (0.972,1.645)
Small or middle-sized cities	0.259 (0.142)	3.314	0.069	1.295 (0.980,1.711)
Large-sized cities	0.057 (0.123)	0.214	0.644	1.059 (0.831,1.348)
Coronary heart disease	0.136 (0.124)	1.191	0.275	1.145 (0.898,1.461)
Type of insurance				
Rural medical service	as ref			
Urban medical service	0.204 (0.114)	3.220	0.073	1.227 (0.981,1.533)
Free medical service	0.276 (0.186)	2.210	0.137	1.318 (0.916,1.898)
Type of medication				
None	as ref			
1	0.444 (0.122)	13.319	<0.001	1.560 (1.228,1.980)
2	0.611 (0.122)	24.939	<0.001	1.843 (1.450,2.343)
3	0.795 (0.156)	25.973	<0.001	2.215 (1.631,3.007)
>4	0.978 (0.157)	39.063	<0.001	2.660 (1.957,3.615)
Drinking				
Never	as ref			
Occasional	0.199 (0.168)	1.406	0.236	1.472 (0.878,1.697)
Always	−0.326 (0.165)	3.926	0.048	0.722 (0.523,0.996)
Smoking				
Never	as ref			
Occasional	0.252 (0.151)	2.789	0.095	1.287 (0.957,1.731)
Always	−0.389 (0.151)	6.637	0.010	0.6781 (0.504,0.911)
Annual physical examination last 10 years	−0.240 (0.097)	6.192	0.013	0.786 (0.651,0.950)
Weekly social activities, days				
Never	as ref			
1–3	−0.480 (0.112)	18.235	<0.001	0.619 (0.497,0.711)
4–6	−0.023 (0.222)	0.011	0.916	0.977 (0.633,1.509)
Every day	−0.775 (0.125)	38.515	<0.001	0.461 (0.361,0.588)
Weekly exercise, days				
Never	as ref			
1–3	−0.095 (0.120)	0.625	0.429	0.909 (0.718,1.151)
4–6	0.015 (0.214)	0.005	0.945	1.015 (0.668,1.542)
Every day	−0.343 (0.122)	7.962	0.005	0.710 (0.559,0.901)
Care from family or friends	−0.439 (0.129)	11.555	0.001	0.645 (0.501,0.831)
Support from family	0.293 (0.098)	8.895	0.003	1.341 (1.106,1.626)
Vision	0.263 (0.093)	8.074	0.004	1.301 (1.085,1.560)
Anxiety	0.190 (0.130)	2.128	0.145	1.209 (0.937,1.562)
Depression	0.688 (0.136)	25.546	<0.001	1.990 (1.524,2.599)

### Analysis of factors affecting the cognitive impairment of non-empty nesters

The dependent variable was whether the non-empty nesters had a combined cognitive impairment or not (assigned values were no = 0, yes = 1), and the statistically significant basic characteristics in Table 1 were used as independent variables in a dichotomous logistic stepwise regression analysis. The results indicated that education level influenced the risk of cognitive impairment, monthly income between RMB 4000 and 6,000 (OR: 0.551, 95% CI: 0.416, 0.731) or more than RMB 6000 (OR: 0.498, 95% CI: 0.379, 0.655), labor income (OR: 0.636, 95% CI: 0.432, 0.937),participating in social activities 1–3 days per week (OR: 0.761, 95% CI: 0.615, 0.942) or every day (OR: 0.625, 95% CI: 0.495, 0.790), care from family or friends (OR: 0.717, 95% CI: 0.564, 0.911) were associated with a lower risk of cognitive impairment. Education level, living in small to medium-sized cities (OR: 1.380, 95% CI: 1.056, 1.803) and large cities (OR: 1.686, 95% CI: 1.344, 2.1161), coronary heart disease (OR: 1.319, 95% CI: 1.046, 1.663), There was concern from family and friends (OR:1.288, 95% CI: 1.076, 1.543), and depression (OR: 1.892, 95% CI: 1.461, 2.451) were also correlated with a higher risk of physical function impairment. Multivariate logistic regression analysis of risk factors for cognitive impairment among adults older adults 60 years or older is presented in Table 5.

**Table 5:** Dichotomous logistic regression analysis of factors influencing cognitive impairment in non-empty nesters.

Influencing factors	*β (SE)*	Wald χ^2^ value	*p* value	Adjusted *OR* (*95%*CI)
Gender	−0.041 (0.099)	0.172	0.678	0.960 (0.791,1.165)
Family income per month, CNY				
<2000	as ref			
2000–4,000	−0.193 (0.124)	2.430	0.119	0.825 (0.647,1.051)
4,000–6,000	−0.596 (0.144)	17.158	<0.001	0.551 (0.416,0.731)
>6,000	−0.697 (0.140)	24.879	<0.001	0.498 (0.379,0.655)
Source of income				
Acquiring from children	as ref			
Retirement income	−0.119 (0.112)	1.126	0.289	0.888 (0.712,1.106)
Labor income	−0.452 (0.198)	5.235	0.022	0.636 (0.432,0.937)
Education level				
Illiteracy	as ref			
Primary school and below	0.421 (0.134)	9.918	0.002	1.524 (1.172,1.980)
Junior high school	2.237 (0.151)	220.429	<0.001	9.368 (6.972,12.586)
Senior high school	1.971 (0.166)	141.742	<0.001	7.181 (5.191,9.935)
College and above	1.821 (0.266)	47.042	<0.001	6.180 (3.672,10.399)
Marital status	−0.199 (0.118)	2.839	0.092	0.819 (0.650,1.033)
Place of residence				
Villages	as ref			
Counties or towns	0.131 (0.133)	0.979	0.323	1.140 (0.879,1.479)
Small or middle-sized cities	0.322 (0.137)	5.552	0.018	1.380 (1.056,1.803)
Large-sized cities	0.532 (0.116)	20.321	<0.001	1.686 (1.344,2.116)
Coronary heart disease	0.277 (0.118)	5.467	0.019	1.319 (1.046,1.663)
Type of insurance				
Rural medical service	as ref			
Urban medical service	−0.168 (0.106)	2.495	0.114	0.846 (0.687,1.041)
Free medical service	−0.102 (0.176)	0.336	0.562	0.903 (0.640,1.275)
Type of medication				
None	as ref			
1	0.111 (0.116)	0.917	0.338	1.117 (0.891,1.401)
2	−0.156 (0.121)	1.653	0.198	0.856 (0.675,1.085)
3	−0.150 (0.150)	0.990	0.320	0.861 (0.641,1.156)
>4	−0.293 (0.151)	3.757	0.053	0.746 (0.555,1.003)
Drinking				
Never	as ref			
Occasional	0.387 (0.248)	2.420	0.120	1.472 (0.904,2.395)
Always	0.265 (0.247)	1.150	0.284	1.303 (0.803,2.113)
Smoking				
Never	as ref			
Occasional	−0.228 (0.144)	2.491	0.115	0.796 (0.600,1.057)
Always	−0.066 (0.137)	0.231	0.603	0.936 (0.717,1.224)
Annual physical examination last 10 years	0.253 (0.092)	7.568	0.006	1.288 (1.076,1.543)
Weekly social activities, days				
Never	as ref			
1–3	−0.273 (0.109)	6.290	0.012	0.761 (0.615,0.942)
4–6	−0.244 (0.208)	1.373	0.241	0.784 (0.521,1.178)
Every day	−0.469 (0.119)	15.556	<0.001	0.625 (0.495,0.790)
Weekly exercise, days				
Never	as ref			
1–3	0.030 (0.119)	0.064	0.800	1.030 (0.817,1.300)
4–6	0.248 (0.206)	1.898	0.168	1.328 (0.887,1.989)
Every day	−0.015 (0.119)	0.015	0.901	0.985 (0.780,1.245)
Care from family or friends	−0.333 (0.122)	7.441	0.006	0.717 (0.564,0.911)
Support from family	−0.034 (0.092)	0.135	0.713	0.967 (0.807,1.158)
Vision	0.349 (0.090)	14.986	<0.001	1.418 (1.188,1.693)
Anxiety	0.092 (0.127)	0.523	0.470	1.096 (0.855,1.405)
Depression	0.638 (0.132)	23.343	<0.001	1.892 (1.461,2.451)

## Discussion

The observed rate of physical function impairment was higher in empty nesters (40.15%) than in non-empty nesters (35.74%), indicating that the former were more vulnerable to physical function impairment than the latter. This result was similar to the data of Cheng et al. (28), who studied 5,570 older adults individuals from the Henan Populations for Epidemiologic Study, but this disability rate was obviously higher than those from the 2015 China Health and Pension Tracking Survey (CHARLS) national survey data (29). The level of disability among empty nesters was significantly higher than that found by other scholars in China. Zhai et al. (30) used the Barthel Index to study the performance of ADLs by older adult rural residents of Liuan City, and found that the ADL impairment rate was 32.36%. Qiao et al. (31) surveyed older adult rural individuals in Jining City and showed that 20.2% had impaired ADL performance. Chen et al. (32) used data from a national baseline survey to study the ADL abilities of older adults in 28 provinces and regions, and the results showed that the rate of ADL impairment was 22.38%. The differences between this study and other scholars’ findings may be due to the different scales used to measure ADL ability in the older adult population and the different populations studied. From the results of our study, combined with a series of scholars’ research reflects, precisely responds to a problem, the incidence of incapacitated older adults in China is at a high level. The incidence of incapacitation in the empty-nested older adults is higher than that in the non-empty-nested older adults in this study, and this study provides a new direction for the older adult population in China, especially focusing on the older adults living alone.

The observed rate of cognitive impairment was higher in empty nesters (27.51%) than non-empty nesters (23.52%), indicating that the former were more vulnerable to cognitive impairment than the latter. The study results were higher than those of some domestic and foreign studies. The results of a domestic meta-analysis that included 25 papers from 2001 to 2018 showed that the prevalence of mild cognitive impairment in older Chinese adults was 14%, including 8% for those older adults 60–69 years, 13.1% for those older adults 70–79 years, and 23.4% for those older adults >80 years (33). The overall prevalence of mild cognitive impairment in older Chinese adults was 15.5% in a population-based study of 46,011 participants (34). A meta-analysis of 25 studies worldwide showed an overall mild cognitive impairment prevalence of 16% (35). A longitudinal study conducted in the United States, Europe, Asia, and Australia showed that the prevalence of mild cognitive impairment ranged from 5.0 to 36.7% (36). A practice guideline in the United States showed that the prevalence of mild cognitive impairment was 6.7% in people older adults 60–64 years, 8.4% in people older adults 65–69 years, 10.1% in people older adults 70–74 years, 14.8% in people older adults 75–79 years, 25.2% in people older adults 80–84 years, and 37.6% in people older adults 85 years and older (37). The aforementioned differences across countries or regions may be related to a combination of factors such as race, lifestyle, economic level, and medical model. The results of all studies suggest that the southern region of China should be the main target for the prevention and control of cognitive impairment, and more attention should be given to the cognitive status of empty nesters. The residential status of older adult individuals should also be considered to provide precise interventions. Further studies should pay more attention to the establishment of interventions and management systems combined with regional characteristics.

The results of this study showed that average monthly income, multiple medication use, care by family and friends, and depression were common influencing factors for the occurrence of physical disability in empty nesters and non-empty nesters. With increasing age, older adults experience a decline in body organ function and an increase in the depreciation of health capital, which leads to disability (38). This study showed that multiple medication use was a risk factor for the occurrence of disabling conditions, with a 3.166-fold risk of disabling conditions in empty nesters and a 2.660-fold risk in non-empty nesters. This study shows that older adults with family support were more likely to have disability, which was inconsistent with some scholars’ studies. Physical health status directly or indirectly affects an individual’s mental health and mental status, while mental health and mental status also affect physical health; thus, older adult individuals who are negative and pessimistic are more likely to experience physical impairment, which can lead to disability (39). The results of this study showed that depressed older adults individuals had higher levels of disability, reaching a 1.710-fold higher level in empty nesters and a 1.990-fold higher level in non-empty nesters. We should always pay attention to the psychological state of the older adult population when providing care and help mediate their emotions so that they can maintain a good state of mind and a positive outlook on life. Notably, anxiety was an independent risk factor for disability among empty nesters.

The results of this study showed that retirement income, annual physical examination for the past 10 years, regular weekly physical activity, and care from family and friends were common protective factors for the occurrence of physical disability in both empty nester and non-empty nesters. Literacy, non-smoking, low alcohol consumption and regular exercise are protective factors specific to the occurrence of disability among non-empty nesters.

The results of this study showed that education level, place of residence and depression were common influencing factors for the occurrence of cognitive impairment in both empty and non-empty nesters. A higher education level was associated with a lower risk of physical function impairment, while the contrary result was shown in cognitive impairment. Previous studies (40) tended to support the viewpoint that education level was considered a protective factor because individuals with more education have more access to health-related services and more resources for knowledge to enhance their disease management abilities. However, in our study, more educated older adults individuals had a higher risk of cognitive impairment, which was inconsistent with most studies (41, 42) investigating the relationship between cultural features and disability. However, the findings of Godinho et al. (43) agreed with our findings, which may be explained by complicated neuropathologic theory and structural and functional changes in the aging brain. A plausible explanation is that older adults with polypharmacy status may have a higher comorbidity burden of chronic conditions, which necessitates frequent engagement with healthcare systems. This recurrent clinical contact creates structured opportunities for implementing cognitive screening protocols, thereby facilitating earlier detection and management of both prodromal stages and modifiable risk factors associated with dementia spectrum disorders. Further research is needed to interpret the relationship. Small- and medium-sized cities were risk factors for declining cognitive function. This was inconsistent with Lu et al. (44), who found significantly lower cognitive function in older rural adults than in older urban adults when they investigated the differences in cognitive function between the two groups. A couple of reasons may explain the difference. First, older rural adults are generally less educated than older urban adults, and lower literacy level was a risk factor for cognitive decline (43). Second, the economic status of older persons in urban areas is higher than that of older persons in rural areas, and objective conditions may lead to a decline in the cognitive function of older persons in rural areas. The results of this study showed that depression was a risk factor for cognitive function. Previous studies have also identified depression as a risk factor for cognitive function in older adults (45, 46). This may be because older adults with higher depression scores are more isolated, do not interact with others, and do not participate in social activities. Additionally, older adults in a depressed state have decreased pleasure, are not interested in many things, and have decreased attention and memory, which affects their cognitive function.

The results of this study show that social activities were protective factors affecting cognitive function. The results of numerous studies have shown that social support and social interaction are protective factors for cognitive function in older adults (47, 48). Social activities reflect not only social support but also socialization; the larger the social network is, the greater the social interactions. Adequate social support can have a positive impact on older adults’ physical and mental health and cognitive function, as they gain cognitive stimulation from social interactions. Income was also a protective factor for cognitive function in older adults, which confirms the research of some scholars (49, 50). In contrast, physical exercise in this study was only statistically significant in households with children; there was no significance among empty nesters. It may be that the sample size was too small to detect a difference and needs to be expanded for further analysis. Second, the empty nesters were from urban areas, and the nature of their work differed from that of rural residents different.

We acknowledge that the study had several limitations. First, participants were only from Guangdong Province. Second, only a cross-sectional survey was conducted; therefore, the results failed to prove a causal relationship between risk factors and disability or cognitive impairments. Third, the survey instrument was limited to scales, and in the future it is hoped to harmonize the definitional categories of disability assessment on an international scale. In the future, in-depth longitudinal studies could further explore the trajectory of changes in physical and cognitive functions of older adult individuals who live with children and those who are empty nesters. Intervention studies for risk factors related to the occurrence of disability and cognitive impairment could further investigate whether interventions can improve or delay the occurrence or progression of disability and cognitive impairment, and findings could contribute to positive aging.

## Conclusion

This study shows that the incidence of disability and cognitive impairment in empty nesters was higher than that in non-empty nesters. Monthly income, multiple medications, family support and depression were common factors influencing disability in empty nesters and non-empty nesters. Anxiety was a unique influencing factor of disability in empty nesters, and education level, place of residence and depression were common risk factors for cognitive impairment in empty nesters and non-empty nesters. Taking medication was a unique risk factor for cognitive impairment in empty nesters, while coronary heart disease and physical examination every 10 years were unique risk factors for cognitive impairment in non-empty nesters. Policy makers should take different targeted measures according to whether the older adults individuals are empty nesters, provide appropriate medical and health services for older adult individuals, and slow the occurrence of disability. This study’s findings provide scientific evidence for interventions aimed at risk factors for disability and cognitive impairment in empty nesters and non-empty nesters. In addition, it helps to develop health management strategies for older people.

### Research contributions

This study establishes an analytical framework for investigating empty-nest health with regional specificity in public health research, with principal contributions manifested in two critical dimensions:

Theoretical innovation: We pioneer a dual-track analytical model elucidating health disparities between empty-nest and non-empty-nest older adult populations in Guangdong Province, a hyper-urbanized region in southern China. This novel framework reveals a dose–response relationship between residential patterns and geriatric health outcomes. The quantified disparities establish critical baseline parameters for formulating age-specific health policies in rapidly urbanizing developing nations.

Policy implications: The research addresses a critical knowledge gap regarding mechanisms driving older adult health inequalities during urbanization processes in developing economies. The framework demonstrates particular relevance for urban agglomerations in southern China and comparable high-speed urbanization contexts. Our findings provide an essential evidence base for implementing the “Healthy China 2030” health promotion strategy, advocating a paradigm shift in public health interventions from population-wide approaches to residence-pattern-specific precision strategies. While maintaining universal healthcare coverage principles, this methodology enables targeted resource allocation for vulnerable older adult subgroups.

## Data Availability

The original contributions presented in the study are included in the article/supplementary material, further inquiries can be directed to the first author, JG (jinhuaguo@whu.edu.cn).
